# The Book World of Medicine and Science

**Published:** 1906-10-06

**Authors:** 


### THE BOOK WORLD OF MEDICINE AND SCIENCE.

Surgery: its Theory and Practice. By William Johnson Walsham, F.R.C.S. Eng., M.B. and C.M. Aberd. Formerly Surgeon and Lecturer on Surgery, St. Bartholomew's Hospital, and Member of the Court of Examiners, Royal College of Surgeons, England. 9th edition. Edited by Walter George Spencer, M.S., M.B. (Lond.), F.R.C.S.(Eng.). (J. and A. Churchill, London.)

There are complaints in these days that the serious worker does not produce popular work. If serious work is never popular, it is difficult to understand how this treatise by Walsham—"Surgery: its Theory and Practice"—has reached its ninth edition. The first edition appeared in 1887, and at once took its place as a first favourite on the student's bookshelf. The next edition was better than the first, and naturally its success exceeded all previous issues. In the present (ninth) edition a still greater success is assured. Alterations and additions have been made in the text throughout. Many sections have been rewritten, and to give space for the description of modern advances many of the older methods (now discontinued) have been omitted. Many well-known men have lent their assistance in its compilation and revision, and the editor, Mr. Walter G. Spencer, specially indicates Mr. Jessop, Mr. Cumberbatch, and Dr. Wyatt Wingrave as having helped in bringing the volume to its present perfection. Suggestions have been made by the late Mr. Christopher Heath and by Dr. F. Norman. Dr. Risien Russel assisted in cerebral localisation, and Dr. Purves Stewart rendered help on the subject of spinal disease. There can be no doubt that the book will continue to hold its place in the affections of the students and of all medical men who desire a faithful and reliable guide to modern surgery.

